# Supplementary material for: Real-time imaging of standing-wave patterns in microresonators
Source: Proc Natl Acad Sci U S A. 2024 Feb 27;121(10):e2313981121. doi: 10.1073/pnas.2313981121 (PMC10927573; doi:10.1073/pnas.2313981121)
Supplement: Supplementary file 1 — Appendix 01 (PDF) [file pnas.2313981121.sapp.pdf]

PNAS

Supporting Information for
Real-time imaging of standing-wave patterns in microresonators

Haochen Yan[1,2], Alekhya Ghosh[1,2], Arghadeep Pal[1,2], Hao Zhang[1], Toby Bi[1,2], George Ghalanos[1], Shuangyou Zhang[1], Lewis Hill[1,3], Yaojing Zhang[1], Yongyong Zhuang[1,4], Jolly Xavier[1,5], Pascal Del'Haye[1,2]*
[1]*Max Planck Institute for the Science of Light, Staudtstr. 2, 91058, Erlangen, Germany*
[2]*Department of Physics, Friedrich Alexander University Erlangen-Nuremberg, 91058, Germany*
[3]*SUPA and Department of Physics, University of Strathclyde, 107 Rottenrow, Glasgow G4 0NG, United Kingdom*
[4]*Electronic Materials Research Laboratory, Key Laboratory of the Ministry of Education & International Center for Dielectric Research, School of Electronic Science and Engineering, Faculty of Electronic and Information Engineering, Xi'an Jiaotong University, Xi'an 710049, China*
[5]*SeNSE, Indian Institute of Technology Delhi, Hauz Khas, New Delhi, India*

*Corresponding author: Pascal Del'Haye
Email: pascal.delhaye@mpl.mpg.de

This PDF file includes:

Legends for Movies S1 to S3

Other supporting materials for this manuscript include the following:

Movies S1 to S3

Movie S1 (separate file). Recorded video of scattered light while continuously scanning the laser frequency.

Movie S2 (separate file). Recorded video of scattered light with continuously modulated phase of the standing wave. The fiber stretcher is modulated with a triangle function at 3 Hz and 0.3 V peak-to-peak voltage.

Movie S3 (separate file). Recorded video of scattered light with continuously modulated phase of the standing wave. The fiber stretcher is modulated with a triangle function at 5 Hz and 0.3 V peak-to-peak voltage.
